# Antimicrobial Resistance in the WHO African Region: A Systematic Literature Review 2016–2020

**DOI:** 10.3390/antibiotics13070659

**Published:** 2024-07-17

**Authors:** Laetitia Gahimbare, Claude Mambo Muvunyi, Nathalie Aya Kouadio Guessennd, Jean Pierre Rutanga, Pierre Gashema, Walter Fuller, Ambele Judith Mwamelo, Sheick Oumar Coulibaly, Fausta Shakiwa Mosha, Olga Perovic, Hassiba Tali-Maamar, Ali Ahmed Yahaya

**Affiliations:** 1 World Health Organization Regional Office for Africa Cité du Djoué, Brazzaville P.O. Box 06, Congo; wfuller@who.int (W.F.); mwameloa@who.int (A.J.M.); coulibalysh@who.int (S.O.C.); moshas@who.int (F.S.M.); aliahmedy@who.int (A.A.Y.); 2 AMR Consultants, Kigali P.O. Box 3286, Rwanda; claude.muvunyi@rbc.gov.rw (C.M.M.); guessennd@yahoo.fr (N.A.K.G.); 3 Rwanda Biomedical Center, Kigali P.O. Box 7162, Rwanda; 4 UFR des Sciences Médicales, Université Félix Houphouet Boigny-Institut Pasteur de Côte d’Ivoire, Abidjan P.O. Box, 1563, Côte d’Ivoire; 5 CHU de Québec-Université Laval, L’ Hôtel-Dieu de Québec, Laboratoire de Microbiologie, Québec City, QC G1R 2J6, Canada; rutanga@chudequebec.ca; 6 Repolicy Research Centre, Kigali P.O. Box 7584, Rwanda; p.gashema@sms.ed.ac.uk; 7 College of Medicine and Veterinary Medicine, The University of Edinburgh, Edinburgh EH8 9YL, UK; 8 WHO Collaborating Centre for AMR, National Institute for Communicable Diseases (NICD), a Division of National Health Laboratory Service, Johannesburg 2192, South Africa; olgap@nicd.ac.za; 9 Institut Pasteur d’Algerie, Algiers P.O. Box 16000, Algeria; htalimaamar@pasteur.dz

**Keywords:** antimicrobial resistance, Africa, bacteria, systematic review

## Abstract

Antimicrobial resistance (AMR) is a significant global public health threat. This review presents the most recent in-depth review of the situation of the main AMR types in relation to the most commonly prescribed antibiotics in the World Health Organization (WHO) African Region. Underlying genes of resistance have been analyzed where possible. A search to capture published research data on AMR from articles published between 2016 and 2020 was done using PubMed and Google Scholar, with rigorous inclusion/exclusion criteria. Out of 48003 articles, only 167 were included. Among the tested gram-negative bacteria species, *Klebsiella* spp. remain the most tested, and generally the most resistant. The highest overall phenotypic resistance for imipenem was reported in *E. coli*, whereas for meropenem, *E. coli* and *Haemophilus* spp. showed an equal resistance proportion at 2.5%. For gram-positive bacteria, *Streptococcus pneumoniae* displayed high resistance percentages to trimethoprim/sulfamethoxazole (64.3%), oxacillin (32.2%), penicillin (23.2%), and tetracycline (28.3%), whereas *Staphylococcus aureus* contributed to 22.8% and 10% resistance to penicillin and oxacillin, respectively. This review shows that AMR remains a major public health threat. The present findings will help public health decision-makers in developing efficient preventive strategies and adequate policies for antibiotic stewardship and surveillance in line with the global action plan for AMR.

## 1. Introduction

Antimicrobial resistance (AMR) is defined as the inherited or acquired ability of a microorganism to change over time and no longer respond to medicines, making infections harder to treat and increasing the risk of disease spread, severe illness, and death [1,2], AMR remains a significant threat to the treatment of microbial infections globally and, most importantly, in low- and middle-income settings, including Africa [3]. The AMR threat adds to the existing higher burden of bacterial infections in such settings and low access to adequate diagnostics, specifically at intermediate and peripheral levels of the health system [4].

The main factors exacerbating AMR in Africa include the limited supply or access to antimicrobial drugs, while those that are available might be of poor quality or counterfeit [5]. In addition, in low-resource settings such as most of Africa, antimicrobials including antibiotics can be used in an unrestricted manner such as over-the-counter prescriptions and self-medication, and in feeding animals as prophylaxis or growth promoters; all these factors predispose people to AMR [6,7,8]. The limited enforcement of regulations and quality control of drugs may be exacerbated due to poor infection prevention and control (IPC) and water, sanitation, and hygiene (WASH) interventions, and it can accelerate the spread of drug-resistant microorganisms [2,9]. Only a few reports and publications are available on the problem of AMR on the continent. Three main review articles have been published since 2001, and two of them were conducted on the broad clinically relevant bacteria [10,11], and one assessed the issue of AMR more specifically in children with sepsis not only in Africa but also across all other resource-limited countries worldwide [12]. The coordination and implementation of policies to assess and monitor the situation of AMR in Africa is weak despite the availability of the Global Antimicrobial Resistance Surveillance System (GLASS). GLASS was launched in 2015 at the behest of the Sixty-Eighth World Health Assembly in resolution WHA68.7, with the aim of supporting the global action plan on AMR (GAP-AMR), and specifically the second objective of the GAP-AMR, which is to strengthen knowledge through surveillance and research and enhance existing activities [13]. In resource-limited settings, treatment is mostly based on presumptive clinical diagnosis with empirical choice of the antibiotic, but not on antibiotic susceptibility testing (AST) results when they exist. In 2020, the COVID-19 pandemic further contributed to the increased spread of AMR due to the inappropriate use of antibiotics for the case management of patients [14] and a breakdown of antibiotics stewardship and IPC programs [10]. Most low-resource settings are plagued with inadequate infrastructure and a lack of technical skills and essential supplies for the optimal diagnosis and treatment of AMR [15]. This leads to an increase in infectious diseases and associated AMR in low- and middle-income countries (LMICs). The detection of AMR in LMICs is insufficient even in some national reference laboratories, thus reflecting a lack of laboratory and diagnosis capacity [16]. Understanding the recent status of AMR and trends of resistance in the WHO African Region could improve clinical practice by guiding the clinician’s choice of the right antibiotic and informing decision-making for African Region Member States, WHO-AFRO, partners, and stakeholders. This paper systematically reviews the currently available and published data on the etiology of bacterial AMR patterns in the WHO African Region from 2016 to 2020. The analysis focused on the AST methods currently in use, the types of recent AMR patterns, and the regional distribution of resistance patterns. This review report proposes recommendations, future options, and interventions to contain AMR in the WHO African Region. (All abbreviations used in this manuscript are indicated in Supplementary Materials, Table S1).

## 2. Results

### 2.1. Data and Article Characteristics

**Figure 1:**
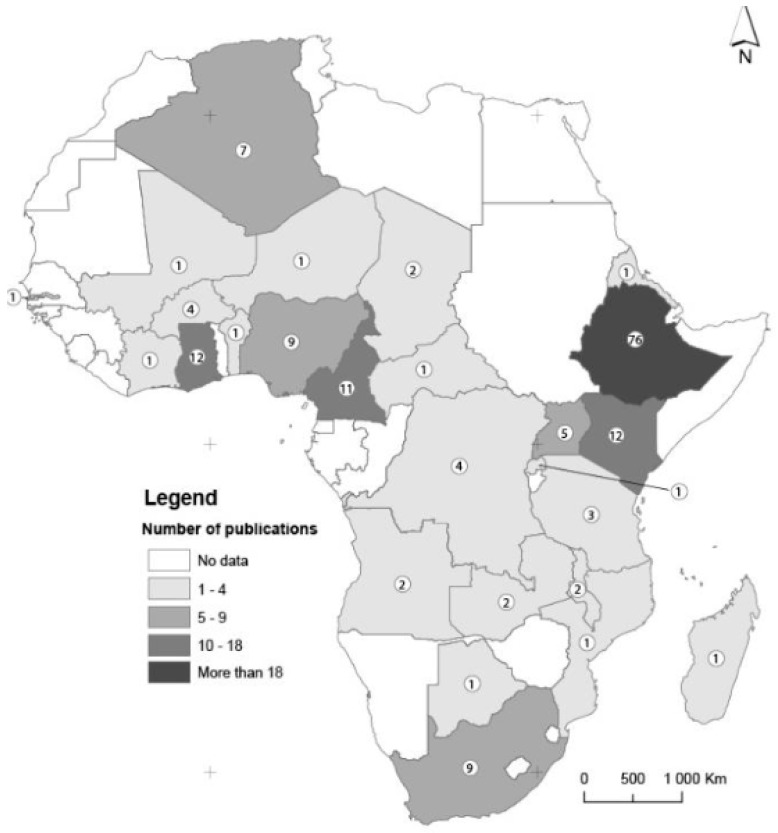
Distribution of articles included in the review by WHOAFR country.

The majority of the final selected articles were published in 2019 (38/167, 22.6%), with most of them having a collection period for the reported isolates falling before 2016 (95/167, 56.5%). As shown in Figure 1 a high number of the reviewed articles were conducted in Ethiopia (76/167, 45.2%) with the rest of the countries represented in a low number of articles; for example, Mali, Niger, and the Central African Republic, etc., are represented in one article (0.6%) (Supplementary Materials, Table S2 and Figure 1 below).

Considering the type of investigation (phenotypic or genotypic and phenotypic), more studies used the phenotypic investigation method (123/167, 73.7%), which mostly relies on the standard microbiological culture, followed by a combination of both phenotypic and genotypic investigations (44/167, 26.3%) (Supplementary Materials, Table S2). For the interpretation of the AST data, most of the studies used the Clinical & Laboratory Standards Institute (CLSI) guidelines (129/167, 76.8%), followed by those of the European Committee on Antimicrobial Susceptibility Testing (EUCAST) (17/167, 10.1%), whereas the CLSI and British Society of Antimicrobial Chemotherapy (BSAC) guidelines were the least used (2/167, 1.2%) or not mentioned in the rest of the articles. In contrast, for the AST methods, the majority of the reviewed studies used disk diffusion (114/167, 67.8%), and for bacterial identification, the majority of the reviewed studies used the common standard for microbiological culture (105/167, 62.5%) (Supplementary Materials, Table S2).

### 2.2. AMR Patterns

Analysis of the AMR results includes three main themes. The first and second themes present reported bacterial AMR patterns from the reviewed articles among gram-negative, and gram-positive bacteria, respectively. The third theme presents the main underlying genetic markers of the phenotypic AMR reported among the common gram-negative and gram-positive bacteria in the WHO African Region.

#### 2.2.1. Theme 1: AMR Patterns among Gram-Negative Bacteria in the WHO African Region, 2016–2020

**Figure 2:**
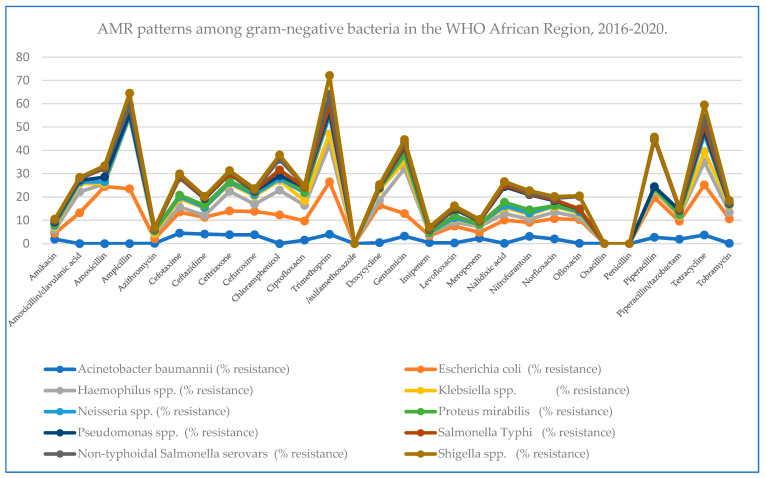
Percentage of AMR patterns among gram-negative bacteria in the WHO African Region, 2016–2020.

Among the commonly reported medical bacteria pathogens, 10 g-negative bacteria species and serovars were selected from all other gram-negative bacteria reported in all the 167 reviewed articles, and their AMR data are presented in (Supplementary Materials, Table S3). They are *Acinetobacter baumannii* (*n* = 2220), *Escherichia coli* (*n* = 1202), *Haemophilus* spp. (*n* = 360), *Klebsiella* spp. (*n* = 1741), *Neisseria* spp. (*n* = 2857), *Proteus mirabilis* (*n* = 8666), *Pseudomonas* spp. (*n* = 1375), *Salmonella* Typhi (*n* = 2000), non-typhoidal *Salmonella* serovars (*n* = 13500), and *Shigella* spp. (*n* = 2500). These gram-negative bacteria were tested against 28 different antibiotics: amikacin, amoxicillin/clavulanic acid, amoxicillin, ampicillin, azithromycin, cefotaxime, ceftazidime, ceftriaxone, cefuroxime, chloramphenicol, ciprofloxacin, trimethoprim/sulfamethoxazole, doxycycline, erythromycin, gentamicin, imipenem, levofloxacin, meropenem, nalidixic acid, nitrofurantoin, norfloxacin, ofloxacin, oxacillin, penicillin, piperacillin, piperacillin/tazobactam, tetracycline, and tobramycin. Among all the 10 tested bacteria species, *Klebsiella* spp. remains the most tested bacteria with a high number of isolates and generally the most resistant. *E. coli* presents most of the reported high AMR percentages (%) for amoxicillin (24.5%), ampicillin (24%), amoxicillin/clavulanic acid (13.2%), chloramphenicol (12.5%), ciprofloxacin (8.2%), and trimethoprim/sulfamethoxazole (22.5%) (Figure 2). The highest overall phenotypic resistance for imipenem is reported in *E. coli*, whereas for meropenem, *E. coli* and *Haemophilus* spp. show an equal resistance proportion at 2.5% (Details are available in Supplementary Materials, Table S3 and also presented in Figure 2 below).

#### 2.2.2. Theme 2: AMR Patterns among Gram-Positive Bacteria in the WHO African Region, 2016–2020

**Figure 3:**
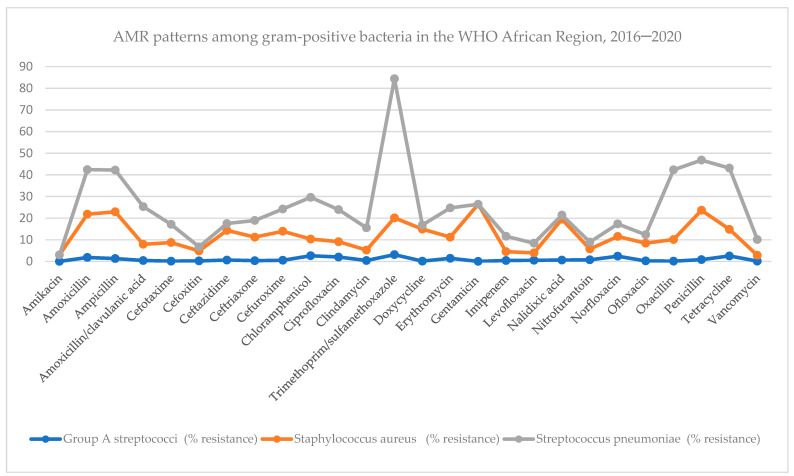
Percentage of AMR patterns among gram-positive bacteria in the WHO African Region, 2016–2020.

Conversely, for gram-positive bacteria (data presented in Figure 3 below and detailed in Supplementary Materials, Table S4), the three main medically important gram-positive bacteria were assayed for antimicrobial susceptibility. These gram-positive bacteria are Group A *streptococci*, *Staphylococcus aureus*, and *Streptococcus pneumoniae*. They were tested against the following 26 different antibiotics: amikacin, amoxicillin, ampicillin, amoxicillin/clavulanic acid, cefotaxime, cefoxitin, ceftazidime, ceftriaxone, cefuroxime, chloramphenicol, ciprofloxacin, clindamycin, trimethoprim/sulfamethoxazole, doxycycline, erythromycin, gentamycin, imipenem, levofloxacin, nalidixic acid, nitrofurantoin, norfloxacin, ofloxacin, oxacillin, penicillin, tetracycline, and vancomycin.

*Streptococcus pneumoniae* shows high resistance percentages against the key tested antibiotics: amoxicillin (20.6%); ampicillin (19.3%); amoxicillin/clavulanic acid (17.4%); chloramphenicol (19.3%); ciprofloxacin (14.8%); trimethoprim/sulfamethoxazole (64.3%); doxycycline (1.9%); erythromycin (1.9%); gentamicin (13.5%); oxacillin (32.2%); penicillin (23.2%); and tetracycline (28.3%).

#### 2.2.3. Theme 3: Genetic Markers Underlying Phenotypic AMR in the WHO African Region

A compilation of the genomic data from the genotypic investigation-based studies shows the genetic markers that are frequently reported to be associated with the common AMR phenotypes among gram-negative and gram-positive bacteria in the WHO African Region. The AMR genes and mutations associated with extended-spectrum β-lactamase (ESBL), metallo-β-lactamases (MBL), carbapenemase, decreased ciprofloxacin susceptibility (DCS), and methicillin resistance are mostly reported among *Klebsiella pneumoniae*, *Pseudomonas aeruginosa*, *Escherichia coli*, *Salmonella Typhi*, and *Staphylococcus aureus*, respectively. For the ESBL genes, *blaCTX-M*, *blaTEM*, and *blaSHV* are highly reported at 522 (60.3%), 203 (51%), and 604 (70%), respectively (Table 1). The same genes are also detected in *Escherichia coli*, but at lower rates compared to *Klebsiella pneumoniae*. The *blaNDM* (90%) among other genes associated with metallo-beta-lactamase production remains the most widely reported and is found at higher frequencies in *Klebsiella pneumoniae*. Furthermore, in gram-negative bacteria, the *blaOXA* (associated with carbapenemase) and gyrA mutations (associated with DCS phenotypes) are reported at 336 (49%) and 487 (25%) in *E. coli* and *S. Typhi*, respectively. Whereas in gram-positive, the mobile genetic element SCCmec, which is associated with MRSA in S. aureus, is reported at 116 (21.4%) (Table 1).

## 3. Discussion

The findings described in this review report are mainly AMR data that have been extracted and analyzed from a total of 167 articles published between 2016 and 2020. For the 167 reviewed articles that constituted this review, it was observed that different panels of antibiotics, AST methods, and AST interpretation guidelines were used. Thus, the standardization of the microbiological identification and AST methods, and the sharing of AMR data, are required to enable regional and international organizations such as the WHO to closely monitor the extent and evolution of the AMR problem in Africa. These AMR cases constitute major and ongoing public health threats; they are also highlighted in the WHO list of critical-priority AMR bacteria for which new research on, discoveries of, and developments in antibiotics are highly needed [3,17,18,19]. Therefore, the timely and continuous surveillance of bacterial infections, as well as the reporting and sharing of AMR data, are needed in Africa to guide the required new approaches for control and the treatment of bacterial infections [3,7,20,21]. In this review, the susceptibility results from selected articles were evaluated with caution, given the inconsistencies in the choice of antibiotic combinations in the various articles. For instance, in some cases, amoxicillin was tested and reported for *Acinetobacter*, and ampicillin and amoxicillin for *Klebsiella* spp., although these bacteria have acquired a natural resistance to the tested antibiotics [22]. In the same trend of natural resistance forms, oxacillin and penicillin were also tested in gram-negative bacteria, although they are known to remain inactive in these bacteria. *Citrobacter* remains an opportunistic bacterium and causes clinical infections among immunocompromised patients [23,24,25]. In all these cases, the compiled, corresponding extracted data were recorded as not applicable, “NA”, in Tables S3 and S4 of the results. Following the analysis of AMR data from the 167 reviewed articles, we observe that resistance in *E. coli*, *Klebsiella pneumoniae*, *Pseudomonas aeruginosa*, *Neisseria* spp., non-typhoidal *Salmonella* serovars, and *Vibrio cholerae* remains significant in Africa [22]. ESBLs’ and carbapenems’ resistance is reported at low but alarming rates in Africa [26]. The results of this review mirror those from previous reviews conducted on AMR in Africa and published in 2017. For instance, the resistance of the key gram-negative bacteria, such as *Enterobacteriaceae*, to the commonly recommended first- and second-line antibiotics is reported in the results of this review, as in the two previous review studies. In addition, for gram-positive bacteria, the resistance of *Staphylococcus aureus* to oxacillin and resistance rates to penicillin in *Streptococcus pneumoniae* observed in the results of this review were previously reported [10,12,27].

This underscores the persistence of the AMR problem in the WHO African Region and calls for the need for a broader use or adoption of tools such as the WHO AWaRe categorization, thus limiting potential abuse or misuse that could further accelerate the selection pressure for increased resistance and result in high morbidity and mortality in the African region. The present findings also confirm the presence of some important genetic markers for the key resistance forms, such as ESBL and carbapenem production, among pathogens causing BSI, STI, enteric fever, and invasive salmonellosis in Africa. It is already known that carbapenem resistance is mediated via transferable carbapenemase-encoding genes [28,29]. These genes are already known from different research projects conducted in Europe, Asia, and South America [30,31,32], whereas the African setting remains less explored and documented [33]. Gram-negative bacteria, mainly *Enterobacteriaceae*, become resistant to carbapenems through three main mechanisms: enzyme production, efflux pumps, and porin mutations [34,35]. Three important groups of enzymes that are responsible for carbapenem resistance are KPC (*Klebsiella pneumoniae* carbapenemase) (Ambler class A), MBLs (Metallo-ß-Lactamases) (Ambler class B) and OXA-48-like (Ambler class D), and *blaIMP*, *blaVIM-1*, *blaSPM-l*, *blaNDM-1*, *blaOXA-23*, *blaOXA-24*, *blaOXA-58* and *blaKPC* resistance determinant genes reported from Africa [36,37]. The results of this review emphasize and recall the urgent need to improve surveillance programs in each and every country of the WHO African Region to support antimicrobial stewardship.

The main limitations of this review include the exclusion of articles and reports published in languages other than English and French. For instance, there are several articles that are published in Spanish and Portuguese. Therefore, there could be articles from Spanish-speaking African countries (Equatorial Guinea) and Portuguese-speaking African countries (Angola, Cabo Verde, Guinea-Bissau, Mozambique, and São Tomé and Príncipe) that were missed. Second, this review cannot guarantee the full representativeness of AST data since it only focused on articles with free access to their full content. In addition, this review reported AMR data only for medically important pathogens that are mostly reported in African laboratory settings. Furthermore, most African countries have poorly functioning laboratories, AMR surveillance, and reporting systems. Therefore, their data were not accessible and were not included in this report.

There were very few reports from South Africa, which has a better-functioning health and national AMR surveillance system than neighboring countries [38,39]. These data were not accessible for the search conducted for this review, and therefore, larger AMR trends might have been missed. A further limitation relates to combining AMR results from different patient groups across different countries to compare the data. This approach might have leveled out peaks in resistance in different settings. This report presents an in-depth review of the most recent situation of AMR to the commonly prescribed antibiotics on the African continent. These AMR cases constitute a major and ongoing public health threat, and they are highlighted on the WHO list of critical-priority AMR bacteria for which new research on, discoveries of, and developments in antibiotics are highly needed. The findings of this review will fill gaps in AMR data for Africa and help decision-makers and healthcare workers develop more efficient preventive strategies, as well as adequate policies for antibiotic stewardship and surveillance, in line with the global action plan for AMR. More timely and effective surveillance studies and programs for bacterial infections are required to deal with the current AMR threats presented in this report for the WHO African Region. This will result in a considerable positive impact on patients and reduce healthcare costs on the continent.

## 4. Materials and Methods

### 4.1. Data Sources and Search Strategy

Two freely accessible scientific web search engines, PubMed and Google Scholar, were searched with the aim of capturing published research data on AMR. The search was extended to the entire African continent and later excluded articles from countries that are not part of the WHO African Region. The following keywords related to the review topic were used: “Antimicrobial Resistance Africa”, “Antimicrobial Susceptibility Africa”, “Surveillance Africa”, “Diagnostic Africa”, and “Bacteria Diagnostic Africa”. These five search keywords were entered into PubMed and Google Scholar, respectively. All articles on AMR in the WHO African Region were then retrieved. The same search strategy was repeated for the second round of the search with the use of the same keywords but with the name of each country of the African Region added next to it for each search.

### 4.2. Selection and Rejection Criteria

Retrieved articles were retained if they met and satisfied the following inclusion criteria: a publication date between 2016 and 2020; a publication language of English or French; reporting AMR research data on humans; having been conducted in countries of the WHO African Region; the free accessibility of their abstracts and full texts; reporting data on AST; providing details on the total number of studied isolates; and a case report or case series format. Conversely, retrieved articles were rejected based on the following exclusion criteria: having been conducted as randomized control trials of antibiotics; surveillance studies of antibiotic use/misuse; molecular investigations of AMR molecular markers; or reviews of given types of AMR. In addition, reference lists of potential research articles retained at this stage were subsequently scrutinized for inclusion criteria, and those that met the criteria were added to the final list of potential research articles to be reviewed and included in this report.

### 4.3. Selection Procedure

**Figure 4:**
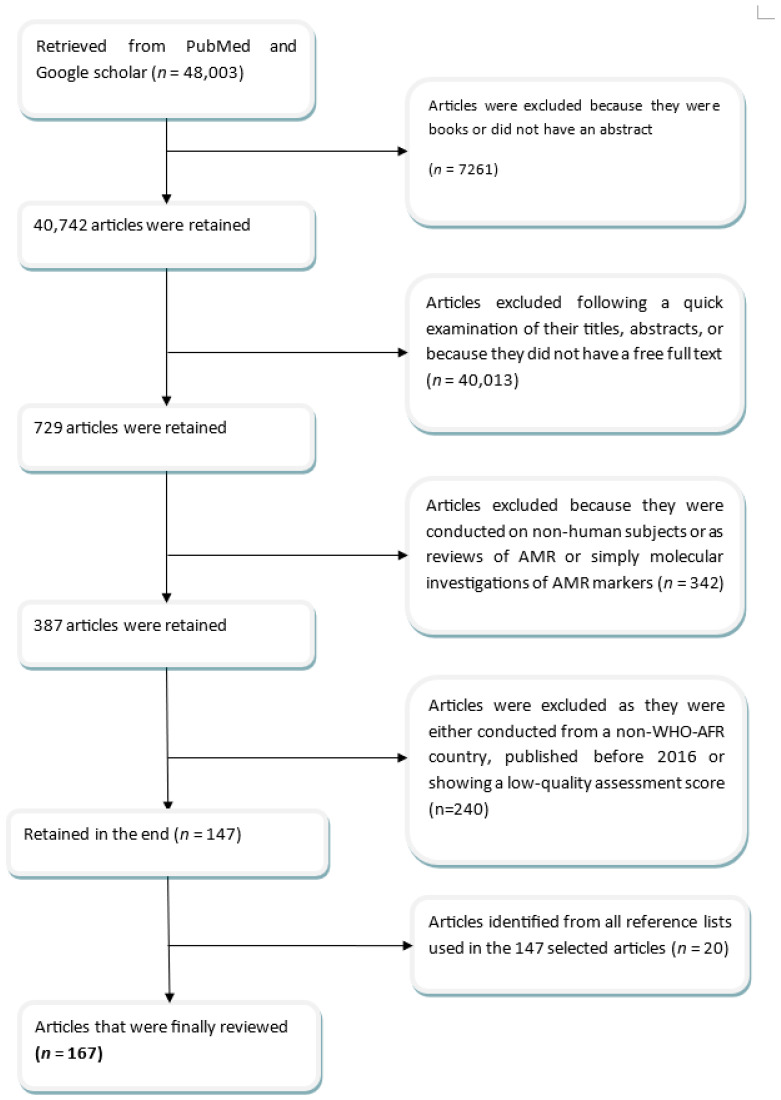
Illustration of the search and selection flow for articles.

From the initially retrieved 48,003 articles, 7261 were excluded because they were either books or did not have an abstract; 40,013 articles were excluded because they did not fit with our review topic or lacked free full-text versions. A total of 342 articles were subsequently excluded because they were conducted on non-human subjects as reviews of AMR or simply as molecular investigations of AMR markers. At the next stage, 240 articles were excluded because they were either published before 2016, showed a low-quality assessment score, or were conducted in a country outside the WHO African Region. A total of 147 articles that were retained were also added to the other 20 that were identified from all their respective reference lists. This yielded a total of 167 articles that were analyzed in this review. A complete description of all the steps followed, as described in the reporting of systematic reviews used to select the final articles that were included in this review, is found in the flowchart presented in Figure 4 below [39].

### 4.4. Article Quality Assessment

The quality of each of the 167 selected articles was assessed based on the methodological quality and appropriateness for inclusion without limiting the consideration to their generated results [40]. The criteria for quality assessment that were followed are the following:

(a) Is the research question clear and adequate for the study?
(b) Is the study design used appropriate to the set research question?
(c) Was the sampling method appropriate for the set research question and design?
(d) Were data collected and managed systematically?
(e) Were the collected data analyzed appropriately?

### 4.5. Data Extraction

The extracted data from the 167 reviewed articles were compiled in an Excel database (Excel 2016) that was designed for the purpose of this review report. The data that were extracted from articles are related to the first author, publication year, title, DOI/PMID/Link, WHO-AFR country, study/data collection period, study objective, study design, study subjects, inpatient or outpatient department, type of sample, age group, reported bacteria, and infection/syndrome. Additionally, the extracted data included the source of infection (healthcare- or community-acquired infection), investigation method (phenotypic or genotypic), bacterial identification method, AST method, tested antibiotics, and AST guidelines. (Details are available in Supplementary File S1).

### 4.6. Data Analysis

The total number of clinical bacterial isolates tested in each selected article was extracted, and the overall number of isolates tested was calculated for susceptibility against key antibiotics. From this step, the percentage of resistant bacteria isolates could then be deduced from the total number of tested isolates for each of the reported bacteria spp.

## 5. Conclusions

This article has presented an in-depth review of the most recent situation of AMR to the commonly prescribed antibiotics on the African continent. These AMR cases constitute a major and ongoing public health threat, and they are highlighted in the WHO list of critical-priority AMR bacteria for which new research on, discoveries of, and developments in antibiotics are highly needed. The findings of this review will fill the gaps in AMR data for Africa and help decision-makers and healthcare workers develop more efficient preventive strategies, as well as adequate policies for antibiotic stewardship and surveillance, in line with the global action plan for AMR. This review has also emphasized the need to conduct surveillance studies that are both timely and effective, aligning with current strategies to combat resistance. These studies should focus on various approaches, including targeting antimicrobial-resistant enzymes and bacteria, developing drug delivery systems, utilizing physiochemical methods, and exploring unconventional strategies and programs for bacterial infections. These efforts are essential to addressing the AMR threats highlighted in this review for the WHO African Region. This will result in a considerable positive impact on patients and reduce healthcare costs on the continent.

## 6. Future Perspectives

Antimicrobial resistance (AMR) is a looming global crisis akin to a pandemic in its scale. To tackle this, in the future, a unified effort is imperative, bolstered via enhanced national action plans and improved laboratory capabilities for testing and research. Implementing antimicrobial stewardship (AMS) programs in African healthcare facilities is crucial to this endeavor, as it effectively regulates antimicrobial use. The present review has highlighted critical gaps in AMR data in the WHO Africa region. Integrating AMR surveillance into routine national health monitoring in Africa is equally vital, requiring dedicated budget allocations. A failure to address AMR could regress us to a pre-antibiotic era. By fortifying AMS initiatives and surveillance practices, we can strive towards the global goal of containing AMR in the future.

## Figures and Tables

**Table 1:** Percentage AMR genetic markers among gram-negative and gram-positive bacteria isolates in Africa, 2016–2020.

Bacteria	ESBL			Metallo-*β*-Lactamase					Carbapenemase	MRSA	DCS	
	*blaCTX-M* *n* (%)	*blaTEM n* (%)	*blaSHV n* (%)	*blaNDM n* (%)	*blaSPM n* (%)	*oprD* *n* (%)	*blaIMP n* (%)	*PSE* *n* (%)	*blaOXA* *n* (%)	*SCCmec n* (%)	*gyrA* Mutation *n* (%)	*gyrB* Mutation *n* (%)
*Klebsiella pneumoniae*	522 (60.3)	203 (51%)	604 (70)	1003 (90%)					242 (43.2)			
*Escherichia coli*	343 (28.8)	191 (43%)	200 (30%)						336 (49)			
*Staphylococcus aureus*										116 (21.4)		
*Salmonella Typhi*											487 (25)	176 (7.8)
*Pseudomonas aeruginosa*					305 (70)	53 (10)	26 (5)	14 (2.5)				

## Data Availability

All data used to support the findings of this review paper are included within this manuscript.

## Supplementary Materials

The following supporting information can be downloaded at: https://www.mdpi.com/article/10.3390/antibiotics13070659/s1, Table S1: Abbreviation table, Table S2: Details of data characteristics, Table S3: Details of percentage of AMR patterns among gram-negative bacteria in the WHO African Region, 2016–2020, Table S4: Details of percentage of AMR patterns among gram-positive bacteria in the WHO African Region, 2016–2020, Supplementary File S1: Additional table showing a list of all 167 papers that were reviewed with some associated data, such as the main author, year of publication, article title, DOI/PMID/Link, WHO African country of publication, study period, study objective, study subjects, bacterial identification method, AST method, tested antibiotics, AST guideline used, etc.
