# Supplementary material for: Antimicrobial Resistance in the WHO African Region: A Systematic Literature Review 2016–2020
Source: Antibiotics (Basel). 2024 Jul 17;13(7):659. doi: 10.3390/antibiotics13070659 (PMC11273377; doi:10.3390/antibiotics13070659)
Supplement: Supplementary file 1 [file antibiotics-13-00659-s001.zip › Supplimentary Tables S1-S4-Revised.pdf]

Title: Antimicrobial Resistance in the WHO African region: A systematic literature review 2016–2020

Table S1. List of Abbreviations

Abbreviation	Meaning
AMR	Antimicrobials resistance
API	Analytical profile index
AST	Antibiotic susceptibility testing
BSI	Bloodstream infection
BSAC	British Society of Antimicrobial Chemotherapy
CAR	Central African Republic
CAMP	Christie–Atkins–Munch-Peterson
CLSI	Clinical & Laboratory Standards Institute
DCS	Ciprofloxacin susceptibility
DOI	Digital Object Identifier
DRC	Democratic Republic of Congo
ESBL	Extended-spectrum β-lactamase
EUCAST	European Committee on Antimicrobial Susceptibility Testing
GAP-AMR	Global action plan on AMR
GLASS	Global Antimicrobial Resistance Surveillance System
IPC	Infection prevention and control
KPC	*Klebsiella pneumoniae* carbapenemase
LMICs	Low- and middle-income countries
MBL	Metallo-β-lactamases
MIC	Minimum inhibitory concentration
MRSA	Methicillin-resistant Staphylococcus aureus
NA	Not Applicable
SCCmec	Staphylococcal cassette chromosome mec
STI	Sexual Transmitted infection
WASH	Water, sanitation and hygiene
WHA	World Health Assembly
WHO	World Health Organization

Table S2. Data Characteristics

**Characteristic**	**Frequency (%)**
Publication year	
2016	25 (14.8)
2017	39 (23.2)
2018	42 (25)
2019	38 (22.6)
2020	23 (13.7)
End data collection period	
Before 2016	84 (50)
Between 2016–2018	65 (38.7)
Between 2019–2020	12 (7.1)
Not mentioned	10 (5.9)
African country	
Algeria	7 (4.2)
Angola	2 (1.2)
Benin	1 (0.5)
Botswana	1 (0.6)
Burkina Faso	4 (2.4)
Cameroun	11 (6.5)
CAR	1 (0.6)
Chad	2 (1.2)
Eritrea	1 (0.6)
Ethiopia	76 (45.2)
Gambia	1 (0.6)
Ghana	12 (7.1)
Ivory Coast	1 (0.6)
Kenya	12 (7.1)

Madagascar	1 (0.6)
Malawi	2 (1.2)
Mali	1 (0.6)
Mozambique	1 (0.6)
Nigeria	9 (5.4)
Rwanda	1 (0.6)
South Africa	5 (2.9)
Tanzania	3 (1.8)
The DRC	4 (2.4)
Uganda	5 (2.9)
Zambia	2 (1.2)
In more than one African country	4 (2.4)
Study design	
Case control	1 (0.6)
Longitudinal	1 (0.6)
Cross-sectional	87 (51.7)
Observational	3 (1.8)
Prospective	12 (7.1)
Retrospective	9 (5.4)
Surveillance	4 (2.4)
Not mentioned	71 (42.3)
Source of Data	
Inpatient	10 (5.9)
Outpatient	8 (4.7)
Both	26 (15.5)
NA	3 (1.7)
Not mentioned	146 (86.9)
Investigation method	
Phenotypic	123 (73.7)

Phenotypic & Genotypic	44 (26.3)
Source of infection	
Hospital acquired	5 (2.9)
Community acquired	4 (2.4)
Both	3 (1.8)
NA	5 (2.9)
Not mentioned	171 (10.1)
AST guidelines	
BSAC	2 (1.2)
CA-SFM	7 (4.2)
CA-SFM & EUCAST	2 (1.2)
CLSI	137 (81.5)
CLSI & BSAC	1 (0.6)
CLSI & EUCAST	8 (4.7)
EUCAST	19 (11.3)
Not mentioned	12 (7.1)
AST Methods	
Broth microdilution	2 (1.2)
Disc diffusion	115 (68.5)
Disc diffusion & broth microdilution	4 (2.4)
Disc diffusion & E-test	12 (7.1)
Disc diffusion & MIC	23 (13.7)
Disc diffusion & VITEK	1 (0.6)
Disc diffusion, D-test & VITEK	1 (0.6)
Disc diffusion, E-test & MIC	2 (1.06)
Disc diffusion, broth microdilution & MIC	1 (0.6)
E-test	4 (2.1)
NA	2 (1.06)
Not mentioned	5 (2.6)

PHX system	1 (0.6)
VITEK	13 (7.7)
VITEK & E-test	1 (0.6)
VITEK & MALDI-ToF	1 (0.6)
VITEK, broth microdilution & E-test	1 (0.6)
Bacterial identification methods	
BACT/ALERT	4 (2.4)
BACT/ALERT & API	1 (0.6)
BACTEC	4 (2.4)
BACTEC & API	3 (1.8)
BACTEC & MALDI-ToF	1 (0.6)
BACTEC, API & VITEK	1 (0.6)
BACTEC, BACT/ALERT & API	2 (1.2)
Conventional BC	1 (0.6)
NA	1 (0.6)
Not mentioned	4 (2.4)
Standard microbiological culture & API	20 (11.9)
Standard microbiological culture & MALDI-ToF	11 (6.5)
Standard microbiological culture	105 (62.5)
Standard microbiological culture & BACTEC	1 (0.6)
Standard microbiological culture & CAMP Test	6 (3.5)
Standard microbiological culture & Hodge test	1 (0.6)
Standard microbiological culture & Vibrio cholera TM Difco BD	1 (0.6)
Standard microbiological culture & VITEK	10 (5.9)
Standard microbiological culture, API & MALDI-ToF	4 (2.4)
Standard microbiological culture, API & VITEK	1 (0.6)
Standard microbiological culture, CAMP test, modified Hodge Test & CIM	1 (0.6)
VITEK	5 (2.9)
CAR: Central African Republic; DRC: Democratic Republic of the Congo; NA: not applicable	

Table S3. AMR patterns among gram-negative bacteria in the WHO African Region, 2016–2020.

**Antibiotic**	*Acinetobacter baumannii* ***n*** **(% resistance)**	*Escherichia coli* ***n*** **(% resistance)**	*Haemophilus spp.* ***n*** **(% resistance)**	*Klebsiella spp.* ***n*** **(% resistance)**	*Neisseria spp.* ***n*** **(% resistance)**	*Proteus mirabilis* ***n*** **(% resistance)**	*Pseudomonas spp.* ***n*** **(% resistance)**	*Salmonella* **Typhi** ***n*** **(% resistance)**	**Non-typhoidal** *Salmonella serovars* ***n*** **(% resistance)**	*Shigella spp.* ***n*** **(% resistance)**
Amikacin	42 (1.9)	30 (2.5)	5 (1.4)	22 (1.3)	20 (0.7)	52 (0.6)	11 (0.8)	14 (0.7)	27 (0.2)	10 (0.4)
Amoxicillin/clavulanic acid	NA	159 (13.2)	32 (9)	61 (3.6)	16 (0.6)	64 (0.7)	NA	16 (0.8)	0 (0)	12 (0.5)
Amoxicillin	NA	294 (24.5)	4 (1.1)	NA	26 (0.9)	184 (2)	NA	79 (3.7)	54 (0.4)	18 (08)
Ampicillin	NA	282 (23.5)	110 (30.8)	NA	12 (0.4)	104 (1.1)	NA	67 (3.2)	36 (0.3)	122 (5.2)
Azithromycin	2 (0.1)	24 (2)	7 (2)	6 (0.3)	28 (1)	8 (0.1)	7 (0.5)	0 (0)	0 (0)	16 (0.7)
Cefotaxime	100 (4.5)	108 (9)	8 (2.2)	61 (3.5)	24 (0.9)	60 (0.7)	98 (7.4)	0 (0)	108 (0.8)	20 (0.9)
Ceftazidime	91 (4.1)	87 (7.2)	3 (0.8)	56 (3.2)	6 (0.2)	80 (0.9)	39 (2.9)	11 (0.5)	63 (0.5)	0 (0)
Ceftriaxone	84 (3.8)	123 (10.2)	30 (8.4)	57 (3.3)	14 (0.5)	54 (0.6)	40 (3)	0 (0)	171 (1.2)	6 (0.3)
Cefuroxime	85 (3.8)	120 (10)	11 (3.1)	61 (3.5)	16 (0.6)	80 (0.9)	6 (0.5)	9 (0.4)	27 (0.2)	14 (0.6)
Chloramphenicol	NA	147 (12.3)	38 (10.6)	72 (4.2)	20 (0.7)	120 (1.3)	NA	53 (2.5)	630 (4.5)	44(1.9)
Ciprofloxacin	34 (1.5)	99 (8.2)	24 (6.7)	34 (2)	94 (3.4)	34 (0.4)	26 (2)	0 (0)	90 (0.6)	0 (0)
Trimethoprim /sulfamethoxazole	90 (4)	270 (22.5)	93 (16.1)	77 (4.5)	224 (8)	142 (1.6)	NA	62 (2.9)	630 (4.5)	186 (8)
Doxycycline	9 (0.4)	192 (16)	8 (2.2)	77 (4.5)	18 (0.6)	10 (0.1)	4 (0.3)	9 (0.4)	63 (0.5)	8 (0.3)
Gentamicin	71 (3.2)	117 (9.7)	69 (19.3)	51 (3)	76 (2.7)	46 (0.5)	39 (2.9)	12 (0.6)	198 (1.4)	30 (1.3)
Imipenem	9 (0.4)	36 (3)	3 (0.8)	6 (0.3)	4 (0.1)	20 (0.2)	16 (1.2)	3 (0.1)	54 (0.4)	16 (0.7)
Levofloxacin	7 (0.3)	87 (7.2)	6 (1.7)	25 (1.4)	10 (0.4)	104 (1.1)	35 (2.6)	10 (0.5)	63 (0.5)	12 (0.5)
Meropenem	51 (2.3)	30 (2.5)	9 (2.5)	11 (0.7)	6 (0.2)	11 (0.1)	17 (1.3)	3 (0.1)	45 (0.3)	10 (0.4)

Nalidixic acid	3 (0.1)	108 (10)	10 (2.8)	45 (2.6)	20 (0.7)	138 (1.5)	88 (6.7)	15 (0.7)	180 (1.3)	4 (0.2)
Nitrofurantoin	70 (3.1)	72 (6)	5 (1.4)	34 (2)	14 (0.5)	132 (1.5)	86 (6.5)	6 (0.3)	72 (0.5)	20 (0.9)
Norfloxacin	44 (2)	105 (8.7)	10 (2.8)	41 (2.4)	10 (0.4)	0 (0)	28 (2.1)	9 (0.4)	99 (0.7)	16 (0.7)
Ofloxacin	3 (0.1)	123 (10.2)	4 (1.1)	27 (1.6)	16 (0.6)	78 (0.9)	2 (0.2)	5 (0.2)	747 (5.3)	8 (0.3)
Oxacillin	NA	NA	NA	NA	NA	NA	NA	NA	NA	NA
Penicillin	NA	NA	NA	NA	NA	NA	NA	NA	NA	NA
Piperacillin	60 (2.7)	204 (17)	10 (2.8)	11 (0.6)	18 (0.6)	16 (0.2)	7 (0.5)	2 (0.1)	90 (0.6)	14 (0.6)
Piperacillin/tazobactam	43 (1.9)	93 (7.7)	8 (2.2)	6 (0.3)	8 (0.3)	6 (0.1)	24 (1.8)	6 (0.3)	45 (0.3)	12 (0.5)
Tetracycline	83 (3.7)	258 (21.5)	35 (9.8)	78 (4.5)	202 (7.2)	156 (1.7)	NA	53 (2.5)	423 (3)	130 (5.6)
Tobramycin	2 (0.1)	126 (10.5)	10 (2.8)	55 (3.2)	6 (0.2)	4 (0.04)	5 (0.4)	7 (0.3)	27 (0.2)	18 (0.8)

Table S4: AMR patterns among gram-positive bacteria in the WHO African Region, 2016-2020

**Antibiotic**	*Group A streptococci* *n* **(% resistance)**	*Staphylococcus aureus* *n* **(% resistance)**	*Streptococcus pneumoniae* *n* **(% resistance)**
Amikacin	NA	1300 (3)	NA
Amoxicillin	25 (1.8)	8800 (20)	1600 (20.6)
Ampicillin	18 (1.3)	9500 (21.6)	1500 (19.3)
Amoxicillin/clavulanic acid	5 (0.4)	3300 (7.5)	1350 (17.4)
Cefotaxime	2 (0.1)	3800 (8.6)	650 (8.4)
Cefoxitin	3 (0.2)	2000 (4.6)	150 (1.9)
Ceftazidime	8 (0.6)	6000 (13.7)	250 (3.2)
Ceftriaxone	4 (0.3)	4800 (10.9)	600 (7.7)
Cefuroxime	7 (0.5)	5900 (13.4)	800 (10.3)
Chloramphenicol	36 (2.6)	3400 (7.7)	1500 (19.3)
Ciprofloxacin	28 (2)	3100 (7.1)	1150 (14.8)
Clindamycin	5 (0.4)	2100 (4.8)	800 (10.3)
Trimethoprim/sulfamethoxazole	43 (3.1)	7468 (17)	5000 (64.3)
Doxycycline	2 (0.1)	6500 (14.8)	150 (1.9)
Erythromycin	20 (1.4)	4300 (9.8)	1050 (13.5)
Gentamicin	NA	2800 (6.4)	NA
Imipenem	5 (0.4)	1800 (4.1)	550 (7.1)
Levofloxacin	7 (0.5)	1500 (3.4)	350 (4.5)
Nalidixic acid	9 (0.6)	8300 (18.9)	150 (1.9)
Nitrofurantoin	10 (0.7)	2200 (5.0)	250 (3.2)
Norfloxacin	34 (2.4)	4000 (9.1)	450 (5.8)
Ofloxacin	3 (0.2)	3600 (8.2)	300 (3.9)
Oxacillin	2 (0.1)	4400 (10)	2500 (32.2)
Penicillin	11 (0.8)	10,000 (22.8)	1800 (23.2)
Tetracycline	35 (2.5)	5400 (12.3)	2200 (28.3)
Vancomycin	2 (0.1)	1200 (2.7)	600 (7.3)
